# Application of entire dental panorama image data in artificial intelligence model for age estimation

**DOI:** 10.1186/s12903-023-03745-x

**Published:** 2023-12-15

**Authors:** Se Hoon Kahm, Ji-Youn Kim, Seok Yoo, Soo-Mi Bae, Ji-Eun Kang, Sang Hwa Lee

**Affiliations:** 1 Department of Dentistry, Eunpyeong St. Mary’s Hospital, College of Medicine, The Catholic University of Korea, 1021, Tongil-ro, Eunpyeong-gu, Seoul, 03312 Republic of Korea; 2 Division of Oral & Maxillofacial Surgery, Department of Dentistry, St. Vincent’s Hospital, College of Medicine, The Catholic University of Korea, 93 Jungbu-daero, Paldal-gu, Suwon-si, Gyeonggi-do Republic of Korea; 3 Unidocs Inc, 272 Digital-ro, Guro-gu, Seoul, Republic of Korea; 4 Department of Artificial Intelligence, Graduate School, Korea University, 145, Anam-ro, Seongbuk-gu, Seoul, Republic of Korea; 5 JINHAKapply Corp, 34 Gyeonghuigung-gil, Jongno-gu, Seoul, Republic of Korea

**Keywords:** Age determination, Artificial intelligence, Forensic dentistry, Panoramic radiography, Deep learning

## Abstract

**Background:**

Accurate age estimation is vital for clinical and forensic purposes. With the rapid advancement of artificial intelligence(AI) technologies, traditional methods relying on tooth development, while reliable, can be enhanced by leveraging deep learning, particularly neural networks. This study evaluated the efficiency of an AI model by applying the entire panoramic image for age estimation. The outcome performances were analyzed through supervised learning (SL) models.

**Methods:**

Total of 27,877 dental panorama images from 5 to 90 years of age were classified by 2 types of grouping. In type 1 they were classified by each age and in type 2, applying heuristic grouping, the age over 20 years were classified by every 5 years. Wide ResNet (WRN) and DenseNet (DN) were used for supervised learning. In addition, the analysis with ± 3 years of deviation in both types were performed.

**Results:**

For the DN model, while the type 1 grouping achieved an accuracy of 0.1016 and F1 score of 0.058, the type 2 achieved an accuracy of 0.3146 and F1 score of 0.2027. Incorporating ± 3years of deviation, the accuracy of type 1 and 2 were 0.281, 0.7323 respectively; and the F1 score were 0.1768, 0.6583 respectively. For the WRN model, while the type 1 grouping achieved an accuracy of 0.1041 and F1 score of 0.0599, the type 2 achieved an accuracy of 0.3182 and F1 score of 0.2071. Incorporating ± 3years of deviation, the accuracy of type 1 and 2 were 0.2716, 0.7323 respectively; and the F1 score were 0.1709, 0.6437 respectively.

**Conclusions:**

The application of entire panorama image data for supervised with classification by heuristics grouping with ± 3years of deviation for supervised learning models and demonstrated satisfactory outcome for the age estimation.

## Background

Age estimation is extremely important in radiographical, clinical and forensic practice. Accurate age estimation is essential for multiple purposes, as it can be applied to determine the precise time and treatment strategy based on clinical findings [1, 2] and it can **serve** as important forensic evidence. In children and adolescents, despite several limitations, the development of dentition is one of the most stable and important markers for age estimation [3, 4]. Compared to other skeletal age evaluations, tooth growth and development are less affected by environmental circumstances [5, 6]. This may be related to the precise genetic control of tooth development and eruption [7].

There are many methods for estimating age based on tooth development, eruption, and mineralization stages [8–10]. However, theses usually provide slightly less accurate estimations. Many researchers have created modified methods to improve the accuracy of age estimations, adjusting the numbers for particular races and populations or constructing more complex methods of analysis [11, 12]. Even if there have been various improvements, learning the complicated methods that differ depending on the observer and require the intensive efforts of professionals for estimation analysis can still be challenging. However, with the recent advancements in deep learning technology, such as neural networks, multiple layers of interconnected nodes can process vast amounts of data. These networks adjust the weights and biases of the nodes to minimize the error between the predicted output and the actual output [13–16].

However, most previous machine learning studies have been based on the simple application of existing age estimation methods that are limited to using specific teeth or parts of dental panoramic images for analysis. This study evaluated the application of entire panoramic image data in the deep learning for the age estimation. The outcome performance of age estimation of two supervised learning models, WideResNet,(WRN) and DenseNet (DS) was analyzed.

## Materials and methods

### Ethical approval

This study was conducted in accordance with the guidelines of the World Medical Association Helsinki Declaration for biomedical research involving human subjects. This study was approved by the Institutional Review Board (IRB) and Clinical Data Warehouse (CDW) data review board of The Catholic University of Korea, Catholic Medical Center (XC21WADI0064). Needs for informed consent were waived by the IRB. Data were collected and administered by CDW and the images were exported under the supervision of Enterprise Data Platform (EDP) of The Catholic University of Korea Information Convergence Institute.

### Data collection and classification

**Fig. 1:**
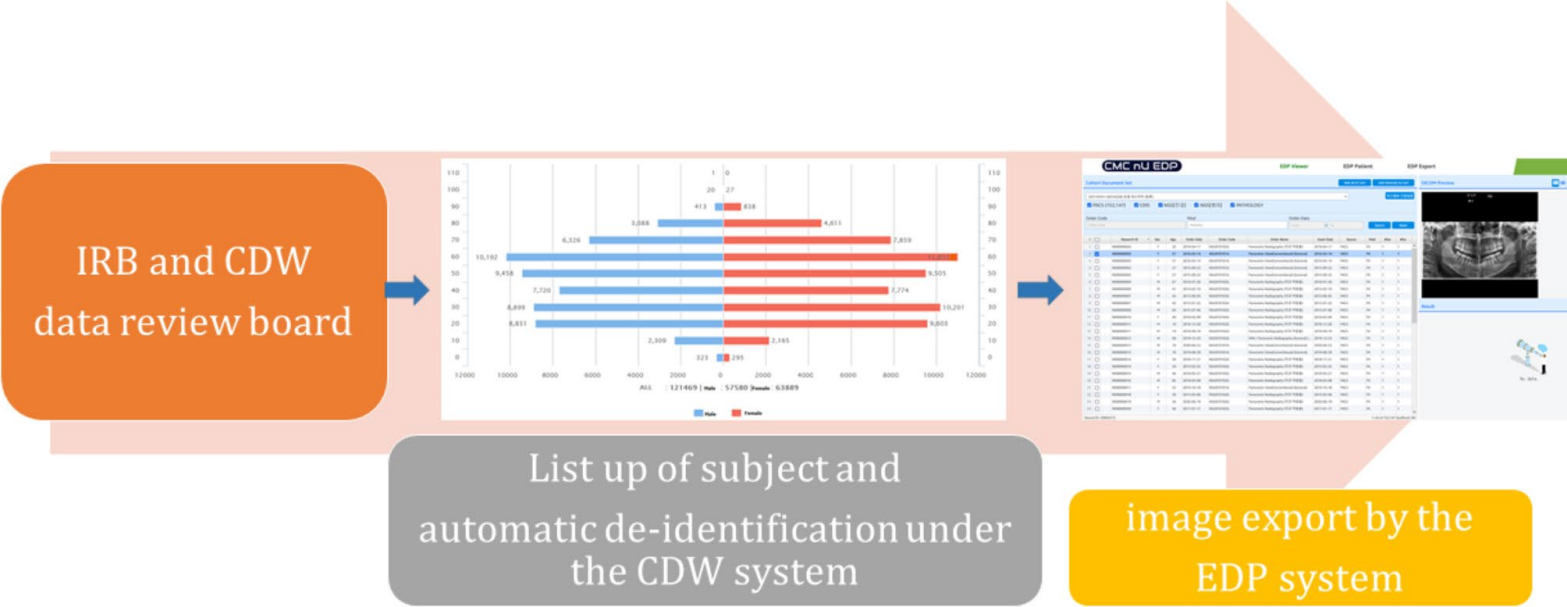
Image data acquisition process through CDW & EDP system

After IRB and Data review board’s approval, the CDW system searched for a list of subjects who visited Eunpyeong St. Mary’s Hospital, St. Vincent Hospital, or Seoul St. Mary’s Hospital of the College of Medicine of The Catholic University of Korea from 2016 to 2020 and underwent panoramic imaging obtained using a ProMax (Planmeca, Helsingki, Finland) or Kodak 8000 Digital Panoramic System (Carestream Health Inc., NY, USA) according to the user manual. The patient data list was undergone to an automatic de-identification process by the CDW system. The panoramic images of listed patients were provided by EDP system after the information had been de-identified and the privacy was ensured. From the collected list, a total of 121,469 qualified panoramic images were downloaded by the EDP system in JPEG format. (Fig. 1) The panorama radiographs with low resolution or pathologic lesion such as cyst and tumors were excluded. Of these radiographs, 27,877 images were randomly selected and labeled from 5 to 90 years of age and gender by two experienced dentists. Each image was resized to 256 × 256 pixels. Since the numbers of instances among classes were unbalanced, a re-sampling technique was utilized to uniformly match the amount of data (Tables 1 and 2).

**Table 1:** Type 1 classification: Numbers of data classified by each age

Numbers of data classified by age and gender in one-year units									
Class name	Number of images	Class name	Number of images	Class name	Number of images	Class name	Number of images	Class name	Number of images
005_F	127	025_F	73	045_F	132	065_F	162	085_F	163
005_M	147	025_M	69	045_M	139	065_M	163	085_M	125
006_F	144	026_F	76	046_F	143	066_F	171	086_F	123
006_M	126	026_M	101	046_M	116	066_M	150	086_M	51
007_F	148	027_F	88	047_F	130	067_F	157	087_F	61
007_M	126	027_M	89	047_M	130	067_M	155	087_M	45
008_F	127	028_F	96	048_F	128	068_F	165	088_F	58
008_M	101	028_M	107	048_M	132	068_M	169	088_M	37
009_F	161	029_F	127	049_F	151	069_F	181	089_F	47
009_M	171	029_M	138	049_M	161	069_M	171	089_M	35
010_F	185	030_F	134	050_F	146	070_F	157	090_F	129
010_M	227	030_M	145	050_M	153	070_M	150	090_M	80
011_F	163	031_F	124	051_F	150	071_F	160		
011_M	179	031_M	143	051_M	132	071_M	154		
012_F	166	032_F	139	052_F	151	072_F	161		
012_M	151	032_M	146	052_M	166	072_M	157		
013_F	173	033_F	144	053_F	171	073_F	165		
013_M	131	033_M	153	053_M	172	073_M	152		
014_F	186	034_F	151	054_F	170	074_F	161		
014_M	176	034_M	158	054_M	167	074_M	148		
015_F	237	035_F	141	055_F	169	075_F	159		
015_M	226	035_M	152	055_M	171	075_M	163		
016_F	199	036_F	138	056_F	178	076_F	162		
016_M	243	036_M	138	056_M	174	076_M	158		
017_F	275	037_F	141	057_F	172	077_F	168		
017_M	277	037_M	141	057_M	166	077_M	150		
018_F	533	038_F	147	058_F	172	078_F	155		
018_M	644	038_M	153	058_M	166	078_M	162		
019_F	909	039_F	135	059_F	167	079_F	157		
019_M	592	039_M	135	059_M	178	079_M	156		
020_F	583	040_F	133	060_F	175	080_F	156		
020_M	467	040_M	156	060_M	180	080_M	151		
021_F	83	041_F	151	061_F	176	081_F	168		
021_M	104	041_M	168	061_M	173	081_M	160		
022_F	78	042_F	164	062_F	173	082_F	152		
022_M	66	042_M	147	062_M	161	082_M	163		
023_F	75	043_F	129	063_F	165	083_F	170		
023_M	63	043_M	126	063_M	156	083_M	159		
024_F	70	044_F	137	064_F	166	084_F	162		
024_M	86	044_M	136	064_M	162	084_M	149		
Sum	27,877								

**Table 2:** Type 2 classification Number of images by age and gender in heuristics grouping where the age over 20 years were classified by every 5 years

Class name	Number of images	Class name	Number of images	Class name	Number of images	Class name	Number of images	Class name	Number of images
005_F	127	011_F	163	017_F	275	31–35_F	699	69–75_F	1144
005_M	147	011_M	179	017_M	277	31–35_M	752	69–75_M	1095
006_F	144	012_F	166	018_F	533	36–40_F	694	76–82_F	1118
006_M	126	012_M	151	018_M	644	36–40_M	723	76–82_M	1100
007_F	148	013_F	173	019_F	909	41–47_F	986	83–89_F	784
007_M	126	013_M	131	019_M	592	41–47_M	962	83–89_M	601
008_F	127	014_F	186	020_F	583	48–54_F	1067	90–96_F	129
008_M	101	014_M	176	020_M	467	48–54_M	1083	90–96_M	80
009_F	161	015_F	237	21–25_F	379	55–61_F	1209		
009_M	171	015_M	226	21–25_M	388	55–61_M	1208		
010_F	185	016_F	199	26–30_F	521	62–68_F	1159		
010_M	227	016_M	243	26–30_M	580	62–68_M	1116		
Sum	27,877								

### Modeling and learning

Total of 27,877 dental panorama images labeled from 5 to 90 years of age were classified by 2 types of grouping. In type 1, they were classified by each age and in type 2, using heuristic grouping, the age over 20 years was classified by every 5 years. In addition, the application of ± 3 years of deviation in both types was also analyzed. Dataset was split into three disjoint sets, including a training set, a validation set and a test set consisting of 13,220, 1,653 and 1,653 images, respectively. (Tables 1 and 2)

DN and WRN models were applied for supervised learning. Stochastic gradient descent was used as an optimizer with a learning rate of 0.005, a mini-batch size of 8, a resize of 256 and a momentum of 0.9.

### Performance analysis

The accuracy, sensitivity, precision, and f1 scores were calculated to evaluate the performance of each model. Python programming language (v. 3.7.11), Pytorch (v.1.8.2) and a graphics card (Nvidia Quadro 6000 8GB *2) were used for analysis.

## Results

Tables 3 and 4 show the model performances of DN and WRN. After a total of 13,220 classified panorama images were trained, 1,653 images were used for validation in each model. The same number of images used for validation was utilized for the test. The best performance was obtained using 40 epochs.

**Table 3:** Performance of DenseNet model

DenseNet						
Number of images		train:13,220, val:1653, test:1653				
parameters		batch8, epoch40, resize256				
performance		Loss	Acc	Precision	Recall	F1-score
Type 1 grouping	Basic prediction	0.5899	0.1016	0.0579	0.0583	0.058
	with ± 3 years deviation	0.5905	0.2813	0.1776	0.1768	0.1764
Type 2 grouping (heuristics)	Basic prediction	0.412	0.3146	0.2115	0.2117	0.2072
	with ± 3 years deviation	0.4116	0.7641	0.6632	0.6658	0.6583

**Table 4:** Performance of WideResNet model

WideResNet							
Number of images		train:13,220, val:1653, test:1653					
parameters		batch8, epoch40, resize256					
performance		Loss	Acc	Precision	Recall	F1-score	
Type 1 grouping	Basic prediction	0.5683	0.1041	0.0598	0.0608	0.0599	
	with ± 3 years deviation	0.5686	0.2716	0.1707	0.1718	0.1709	
Type 2 grouping (heuristics)	Basic prediction	0.4098	0.3182	0.2098	0.2147	0.2071	
	with ± 3 years deviation	0.4091	0.7623	0.6476	0.649	0.6437	

In DN model, the accuracy and F1 score for type 1 grouping were 0.1016 and 0.058, respectively, with a ± 3years of deviation, 0.2813 and 0.1768. For the type 2 grouping, the accuracy and F1 score were 0.3146 and 0.2027, respectively, with a ± 3years of deviation, 0.7641 and 0.6583. The precision and recall score of type 1 grouping were 0.0579 and 0.0583, respectively, with a ± 3years of deviation, 0.1776 and 0.1768. For the type 2 grouping, precision and recall score were 0.2115 and 0.2117, respectively, with a ± 3years of deviation, 0.6632 and 0.6658 respectively.

In WRN model, the accuracy and F1 score of type 1 grouping were 0.1041 and 0.0599, respectively, with a ± 3years of deviation, 0.2716 and 0.1709. For the type 2 grouping, the accuracy and F1 score were 0.3182 and 0.2071, respectively, with a ± 3years of deviation. 0.7323 and 0.6437 respectively. The precision and recall score of type 1 grouping were 0.0598 and 0.0608, respectively, with a ± 3years of deviation, 0.1707 and 0.1718. For the type 2 grouping, precision and recall score were 0.2098 and 0.2147, respectively, with a ± 3years of deviation, 0.7623 and 0.6476 respectively.

**Figs. 2:**
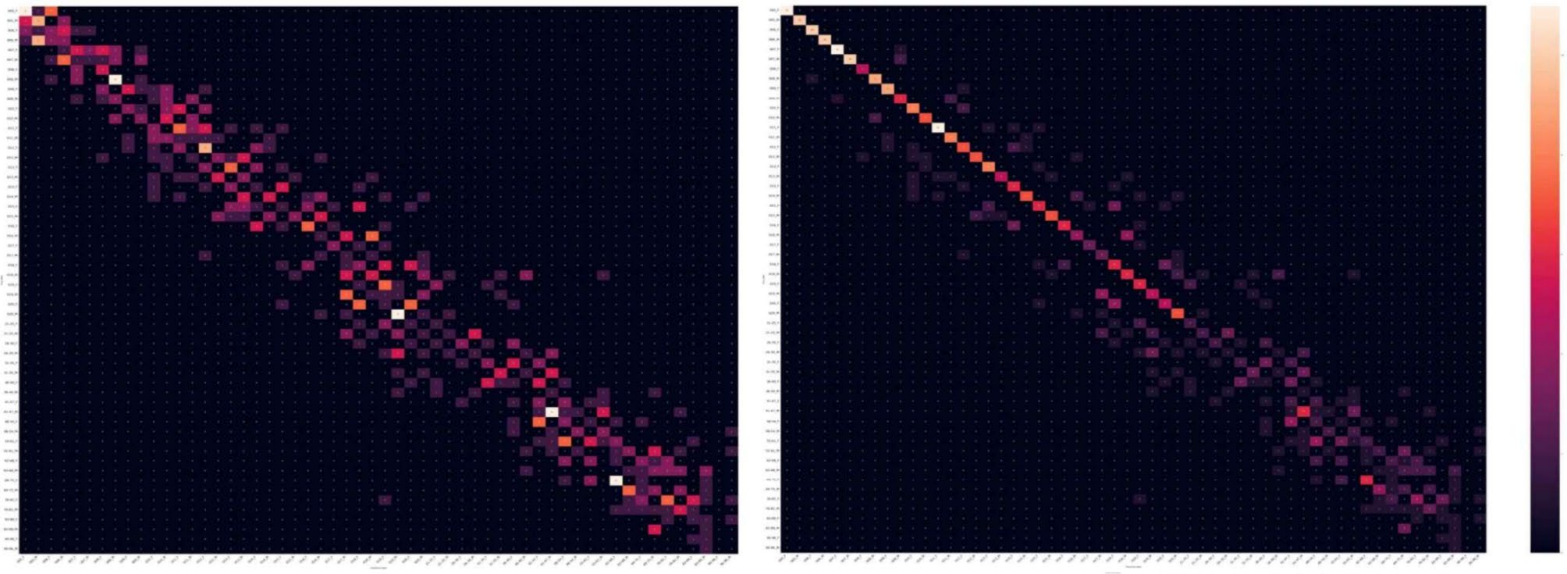
**a** and **b**. Confusion matrix of the results by DenseNet. 2**a** results before heuristic grouping (type1gourping). 2**b**, results after heuristic grouping (type 2 grouping)

**Figs. 3:**
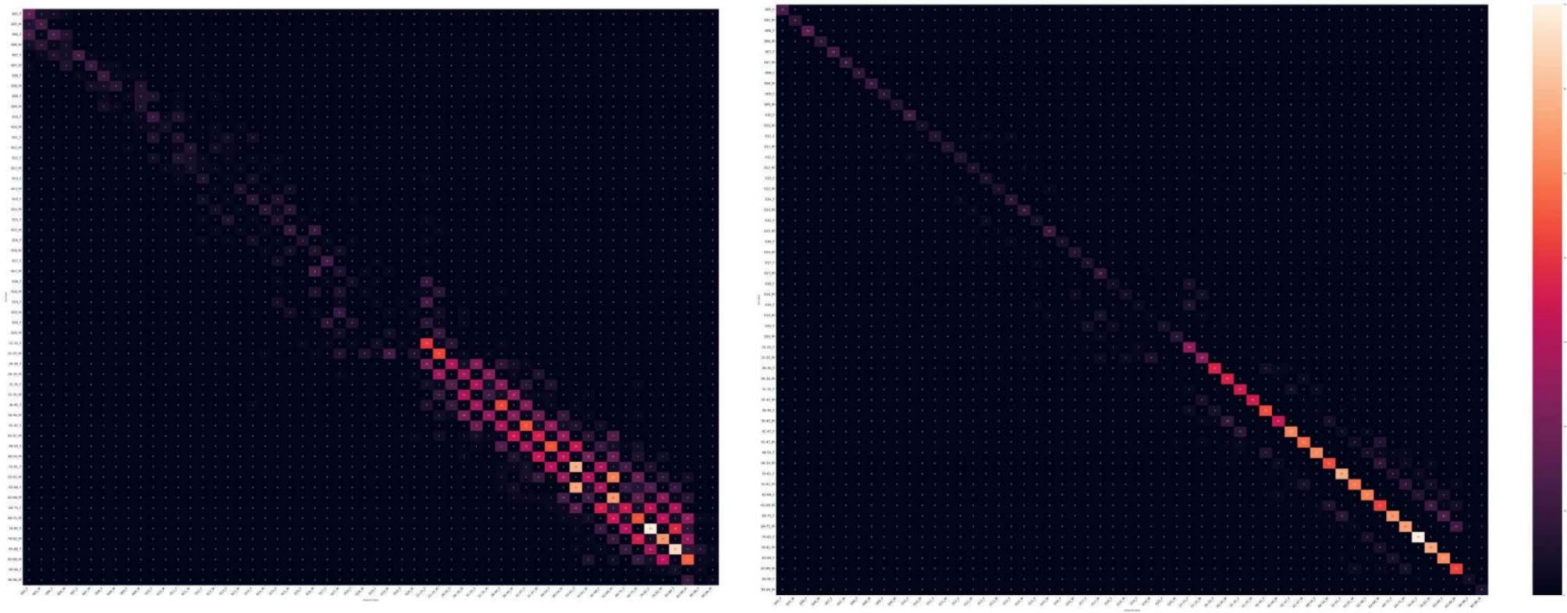
**a** and **b**. Confusion matrix of the results by WideResNet. 3a results before heuristic grouping (type1gourping). 3**b**, results after heuristic grouping (type 2 grouping)

Figures 2 and 3 show the results of both DN and WRN models as a confusion matrix. Considering that a higher the diagonal value of the confusion matrix indicates a more accurate predictive model, the figure present a significant accurate diagnosis in type 2 grouping with a ± 3years of deviation in both DN and WRN models.

## Discussion

Over the years age estimation through imaging has been a well-established method within the field of forensic dentistry, garnering widespread recognition for its inherent utility. Panchbhai discussed various radiological methods used for human age identification. The literature survey identified 46 relevant articles that highlighted the significance of radiography in assessing the extent of dental tissue calcification, crown and root formation, eruption stages, and their correlation with age [17]. Radiographic and tomographic techniques are cost-effective and important tools in forensic dentistry for human identification, especially when combined with information technology resources. Imaging, clinical, and forensic dentists should consider the available methods and legal requirements to ensure accurate age estimation.

Most available age estimation methods are statistical methods that require effort and time during preprocessing measurement. For example, age can be predicted using a regression formula with tooth-coronal index (TCI) [18–20]. In comparison, the present study estimated age based on the overall appearance of a panoramic image rather than the tooth shape, such as measuring the TCI of a specific tooth. The method used in this study differed from previous papers. Simply classifying the images by age reduced the effort of preprocessing step that traditionally required labelling of specific structure of tooth by professionals. And the application of deep learning allowed the process of the data from full panorama images for the analysis of the age estimation not limiting in only from specific teeth data. However, Due to their complexity, AI systems have been often regarded as black boxes, which do not provide any feedback on why and how they arrive at their predictions. In future, efficient application of “explainable AI” is expected to visualize, interpret, and explain the logic behind AI solutions and provide clear prediction strategies [21].

Several other methods for age estimation have been devised. In a machine learning study using Cone beam computed tomography (CBCT) images, the buccal alveolar bone levels of 150 images were utilized by dividing ages of 20–69 years old into 5-year units. In Saric’s CBCT based study, the Random Forest classifier achieved a correlation coefficient of 0.803 and a mean absolute error of 6.022 [22]. However, since the CBCT study used a small number of samples, additional research is needed to determine whether it can be widely applied. In addition, it is more difficult to obtain a CBCT image than a dental panorama for age estimation, and there is a risk of radiation exposure. The present method achieved relatively precise age estimations through heuristic grouping with of supervised classification learning models with 13,220 whole panoramic images.

An AI-based age estimation study using 1,922 panoramic images of patients 15–23 years old was conducted in Malaysia [23]. The study used a hybrid model of convolutional neural networks (CNN) and K nearest neighbors (KNN). Although the method age range was narrow, it successfully estimated age in one-year, six-month, three-months and one-month range with accuracies of 99.98%, 99.96%, 99.87% and 98.78%, respectively. The hybrid (HCNN-KNN) model made good predictions but is based on relatively certain eruption and developmental stages in adolescents and young adults except for those receiving orthodontic treatment, those with dysplasia or those who experienced trauma. The present study was analyzed not only young age patient, but also adult and older patients were included. The machine learning covered the images of the living patient of the age from 5 to 90.

In a CNN study using panoramic photos of 4,035 patients aged 19–85 years in Croatia, age estimation studies were conducted in four groups: 0–15 years old, 16–30 years old, 31–60 years old and over 61 years old with the VGG16 AI learning method [24] through whole orthopantomographic images of archaeological skull. The study demonstrated 73% accuracy. In Korea, a study was conducted on artificial intelligence learning using CNN on 1,586 dental panoramic X-rays [25]. The image of the first molar was exported and the age was estimated by CNN learning. Based on the data from the 10-year-old group, the patients were reclassified into three groups of 0–19 years old, 20–49 years old and 50 years old or older with an estimated accuracy ranging from 89.05 to 90.27%. In both studies, the use of CNN with graphics was attempted rather than simple AI learning and the Korean study also presented the results of heatmap and Grad-CAM. In the present study, grouping was conducted through artificial intelligence learning and the accuracy and f1 score were improved after heuristic grouping. While previous studies have focused on improving accuracy using a wide age range of patients, In the present study, heuristics grouping for over 20 years of age dividing by every 5 years with ± 3 years of deviation for the analysis was applied for provide improve accuracy of age estimation in narrower age range.

It is a known fact that, the external validation using panoramic radiograph datasets from other institutions is necessary to obtain reliable results [26]. However, since each medical imaging data contains private personal information, such data are primarily protected and locked. and not easily accessible and shareable between different institutions due to medical ethical issue [27]. Nevertheless, this study is characterized by the utilization of data from three hospitals of our university located in different districts and with different panorama equipment system. The collection and de-identification of the data were performed using CDW system. And the panorama image files were downloaded and protected by the EDP system of our institution. It would contributed to diminish the overfitting.

The supervised machine learning model used in this study, were WRN and DN. The WRN model is a type of SL using a novel network with decreased depth and increased width of residual networks compared to the previous ResNet model [28]. In addition to the effect of dropout in the residual block, WRN provides better performance and faster training compared to previous deep learning networks, achieving new state-of-the-art and significant improvements compared to ImageNet [28]. While WRN focused on the width of the network, DN focused on the shortcut connections of ResNet [29]. In previous SL involving ResNet, the Highway network, and ResDrop, only the output of the previous layer was sent to the next layer. In comparison, DN receives the output of many previous layers at once and combines the inputs by concatenation rather than addition [29]. Compared with WRN showing the same performance and similar error rates, DN reported an improvement with approximately two times fewer parameters, suggesting deep supervision as the reason for the improved performance [29]. Both SL models exhibited significantly improved results compared to the previous generation, with similar results between them. Based on this performance, both models are being applied in a wide range of medical research fields, with the possibility of more extensive use in the future [30, 31]. Another study compared age estimation on panoramic radiography using the Kvaal method and machine learning. The study found that machine learning techniques, specifically the XG Boosting Reg classifier, showed higher precision in age estimation (MAE: 4.77) compared to the Kvaal method (MAE: 5.68), indicating that ML can enhance age estimation on panoramic radiographs [32]. The reason for the superiority of various machine learning age estimation methods is that the range/quantity of features or patterns that a human can find in a panoramic image is smaller than the features/patterns that a deep neural network can find. It is also difficult to explain the results of age estimation because it is difficult to know which part of the image the deep neural network looked at to identify the features or patterns. However, if advances in this field continue in the future, more convenient and faster age estimation will provide an opportunity to better understand the principles of analysis using deep neural networks.

Artificial intelligence learning could be a useful solution in forensics fields such as age estimation because it can perform complex tasks that were previously difficult to complete in a faster and more accurate manner. In order to achieve this goal, research should continue to utilize and develop various machine learning methods. In the future, it is essential to conduct research on the application and evaluation of various new methods, including semi-supervised learning or SL using artificial intelligence.

## Conclusion

This preliminary study attempts to utilize entire dental panoramic image data in a deep learning model for age estimation. Instead of traditionally requiring professionals to label specific tooth structures, simply classifying the images by age reduced the effort of the preprocessing step. The application of deep learning enabled the analysis of age estimation using data from full panoramic images, rather than being limited to specific teeth data. The performances of both DN and WRN models, with heuristics grouping (where ages over 20 years were classified in 5-year intervals) and a deviation of ± 3 years, yielded satisfactory results in accuracy, recall, precision, and F1 scores. These results are comparable to previous studies on age estimation using traditional methods that require intensive professional effort for analysis and utilize partial data from images, such as teeth. Further clinical and transdisciplinary studies in the medical and advanced technological fields are needed to enhance the quality and simplify the process of age estimation through AI. In the future, the application of AI is expected to assist humans in clinical and dentomaxillofacial radiology fields.

## Data Availability

The datasets generated and/or analyzed during the present study are not publicly available as ethics approval was granted on the basis that only the researchers involved in the study could access the identified data but are available and accessible from the corresponding author on reasonable request.

## Abbreviations

AI: Artificial Intelligence
SL: Supervised Learning
WRN: WideResNet
DS: DenseNet
IRB: Institutional Review Board
CDW: Clinical Data Warehouse
EDP: Enterprise Data Platform
TCI: tooth-coronal index
CBCT: Cone beam computed tomography
CNN: convolutional neural networks
KNN: K nearest neighbors
VGG16: Very Deep Convolutional Networks for Large-Scale Image Recognition
